# Nonstationary Influence of El Niño on the Synchronous Dengue Epidemics in Thailand

**DOI:** 10.1371/journal.pmed.0020106

**Published:** 2005-04-26

**Authors:** Bernard Cazelles, Mario Chavez, Anthony J McMichael, Simon Hales

**Affiliations:** **1**CNRS UMR 7625, Ecole Normale SupérieureParisFrance; **2**IRD UR GEODESBondyFrance; **3**LENA-CNRS UPR 640, CHU Pitié-SalpêtrièreParisFrance; **4**National Centre for Epidemiology and Population Health, Australian National UniversityCanberra, Australian Capital TerritoryAustralia; University of MichiganUnited States of America

## Abstract

**Background:**

Several factors, including environmental and climatic factors, influence the transmission of vector-borne diseases. Nevertheless, the identification and relative importance of climatic factors for vector-borne diseases remain controversial. Dengue is the world's most important viral vector-borne disease, and the controversy about climatic effects also applies in this case. Here we address the role of climate variability in shaping the interannual pattern of dengue epidemics.

**Methods and Findings:**

We have analysed monthly data for Thailand from 1983 to 1997 using wavelet approaches that can describe nonstationary phenomena and that also allow the quantification of nonstationary associations between time series. We report a strong association between monthly dengue incidence in Thailand and the dynamics of El Niño for the 2–3-y periodic mode. This association is nonstationary, seen only from 1986 to 1992, and appears to have a major influence on the synchrony of dengue epidemics in Thailand.

**Conclusion:**

The underlying mechanism for the synchronisation of dengue epidemics may resemble that of a pacemaker, in which intrinsic disease dynamics interact with climate variations driven by El Niño to propagate travelling waves of infection. When association with El Niño is strong in the 2–3-y periodic mode, one observes high synchrony of dengue epidemics over Thailand. When this association is absent, the seasonal dynamics become dominant and the synchrony initiated in Bangkok collapses.

## Abstract 2

In Thailand, dengue transmission patterns are complex, with El Nino showing an association with some epidemics only; other reasons will need to be found for the initiation of other outbreaks

## Introduction

Dengue is a peri-urban disease in the tropics and subtropics, transmitted principally by a single species of mosquito, Aedes aegypti. It has been estimated that 50 to 100 million people each year suffer from dengue and that two-fifths of the human population are at risk. The geographic distributions of dengue and of the potentially fatal form, dengue haemorrhagic fever (DHF), have expanded dramatically in recent decades [1]. The re-emergence of dengue has been connected to societal changes such as population growth, urbanisation, and international travel as well as environmental changes [2,3,4,5,6].

The relationship between climate, human behaviour, and infectious disease is complex, making it difficult to disentangle the different causal mechanisms [3,4,5,6,7,8,9]. It is well established that climate is an important determinant of vector-borne disease epidemics [3,4,5,6,7]. Climate directly influences the biology of the vectors and thereby their abundance and their distribution. Significant correlations have been reported between annual dengue incidence and estimates of Aedes aegypti populations at a national scale, using climate-based models [10]. Meteorological conditions can also directly or indirectly affect pathogen biology and epidemiological factors. Nevertheless, there is relatively sparse evidence of these climatic influences at interannual scales [7]. There is, however, evidence of a relationship between the timing of dengue epidemics and El Niño in the Pacific Islands [11,12] and in some other countries [13].

It is clear that several factors can influence the dynamics of vector-borne diseases, including environmental and climate factors, host–pathogen interactions, and population immunological factors [14]. It has been suggested that the effects of climate are unlikely to contribute to the timing of dengue epidemics in Thailand [14]. Using monthly data for Thailand from 1983 to 1997, Cummings et al. [15] identified travelling waves of dengue, initiated in the capital city, Bangkok, but did not investigate the potential influence of climate.

Dengue incidence data show complex nonlinear dynamics, with strong seasonality, multiyear oscillations, and nonstationarity (changes in dominant periodic components over time). These features of the data mean that conventional statistical methods may be inadequate. Using wavelet analysis, we provide here evidence for a nonstationary association between El Niño and the dynamics of dengue in Thailand. Moreover, we emphasise that this nonstationary association has some important implications for the characteristics of the synchronous dynamics of dengue epidemics in Thailand.

We analysed monthly data of DHF in the 72 provinces of Thailand from 1983 to 1997 [15] in relation to climate variables. Fourier analysis has traditionally been used to analyse the relationships between oscillating time series, but this method is not always appropriate when dealing with complex environmental time series. In particular, this approach can neither take into account the often observed changes in the periodic behaviour of such series, nor quantify the potential association between such series [16,17,18,19,20]. In contrast to Fourier analysis, wavelet analysis has been devised to analyse signals with changing spectra and allows the estimation of the spectral characteristics of a time series as a function of time. Wavelet analysis of a time series provides information on the evolution of the periodic components over time [16,17] and allows the quantification of nonstationary association between two time series [18]. To analyse our datasets we computed the following: (i) wavelet decomposition and wavelet power spectra, which determine the significant oscillating modes; (ii) wavelet coherence patterns, which describe local associations in both time and frequency domains; (iii) phase angles, which indicate the sign of the association, either in phase or out of phase [21]; and (iv) the evolution of the periodic components of each series in the most significant mode of oscillation. We have analysed the time series from the 72 provinces individually, but we show here only results for Bangkok versus the rest of Thailand combined. Similar results are observed with the time series from individual provinces (not shown).

## Methods

### The Data

The numbers of DHF cases used in this study are the monthly reports of DHF in 72 provinces of Thailand (see http://www.jhsph.edu/cir/dengue.html or [15]). We analysed two incidence time series from this dataset: the incidence in Bangkok, the capital city, and the averaged incidence for the rest of Thailand. The climatic data are climatic indexes that describe El Niño oscillations: the Nino 3 index and the Southern Oscillation Index (http://www.cgd.ucar.edu/cas/catalog/climind). We have also quantified the association with rainfall and temperature for the corresponding time periods and geographic areas [22]. For the wavelet analyses, the incidence time series were square root transformed and all the series were normalised before comparison.

### The Wavelet Approach

Among the various approaches developed to study nonstationary data, wavelet analysis is probably the most efficient. In particular, this method gives us the possibility of investigating and quantifying the temporal evolution of time series with different rhythmic components (see [19] and [20] in a populational context). Wavelets constitute a family of functions derived from a single function, the “mother wavelet”, ψ_a,τ_(*t*) , that can be expressed as the function of two parameters, one for the time position τ, and the other for the scale of the wavelets *a*, related to the frequency. More explicitly, wavelets are defined as



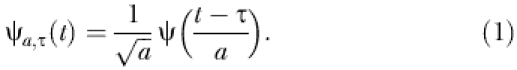



In analysis of “natural signals”, the so-called Morlet wavelet is often applied [16,17]. The Morlet wavelet is defined as



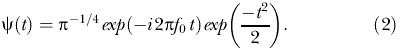



The wavelet transform of a time series *x*(*t*) with respect to a chosen mother wavelet is performed as follows:



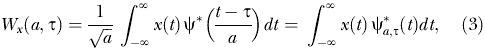



where the asterisk denotes the complex conjugate form. The wavelet coefficient *W_x_*(*a, τ*) represents the contribution of the scale *a* to the signal when time is at different position τ. Computation of the wavelet transform is done along the signal *x*(*t*) simply by increasing the parameter τ over a range of scales *a* until all coherent structures within the signal can be identified.

With the wavelet approach, we can estimate the repartition of variance between scale *a* and different time location τ. This is known as the wavelet power spectrum: *S*_*x*_(*f*,τ) = |*W*_*x*_(*f*,τ)|^2^ . An important point is that the wavelet scale *a* is inversely proportional to the central frequency of the wavelet, *f*_0_. In fact *f* ≈ 1/*a* when *f*_0_ = 2π for the Morlet wavelet. Then scale *a* can be replaced by the frequency *f* or the period *p;* this thus greatly simplifies interpretation of the wavelet analyses. Using the inverse wavelet transform, the original signal can be recovered by integrating the wavelet transform over all scales and locations. This integration can be done over a given periodic band, *p*_1_ to *p*_2_. This allows us to filter the raw signal to obtain its oscillating components in the chosen periodic range.

To quantify statistical relationships between two time series, wavelet coherence can be computed [18]:



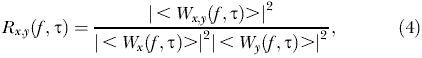



where the angle brackets around terms indicate smoothing in both time and frequency, *W_x_*(*f, τ*) is the wavelet transform of series *x*(*t*), *W_y_*(*f, τ*) is the wavelet transform of series *y*(*t*), and 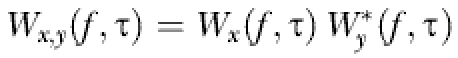 is the cross-wavelet transform. The wavelet coherence provides local information about where two nonstationary signals, *x*(*t*) and *y*(*t*), are linearly correlated at a particular frequency (or period). *R_x,y_*(*f, τ*) is equal to one when there is a perfect linear relationship at a particular time and frequency between the two signals.

In complement to wavelet analysis, we can use phase analysis to characterise the association between signals [21]. The phase difference provides information on the sign of the relationship (i.e., in phase or out of phase). As the Morlet wavelet is a complex wavelet, we can write *W_x_*(*f, τ*) in terms of its modulus, |*W_x_*(*f, τ*)|, and phase,



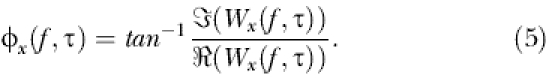



Similarly with the cross-wavelet transform *W_x,y_(f, τ)* one can compute the phase difference:



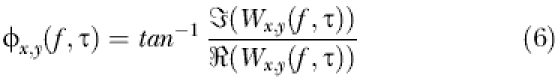



and also the instantaneous time lag Δ*T*(τ) between the time series *x*(*t*) and *y*(*t*). This time lag is computed as



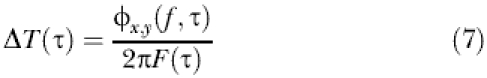



with *F*(τ) the instantaneous frequency defined in a given frequency (or periodic) band:



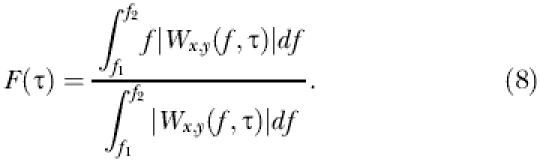



We performed all analyses using original algorithms developed in Matlab (version 6.5, The MathWorks, Natick, Massachusetts, United States). These original algorithms incorporate both cross analyses and adapted statistical procedures (B. Cazelles, M. Chavez, D. Berteaux, F. Ménard, J. O. Vik, et al., unpublished data).

## Results

**Figure 1:**
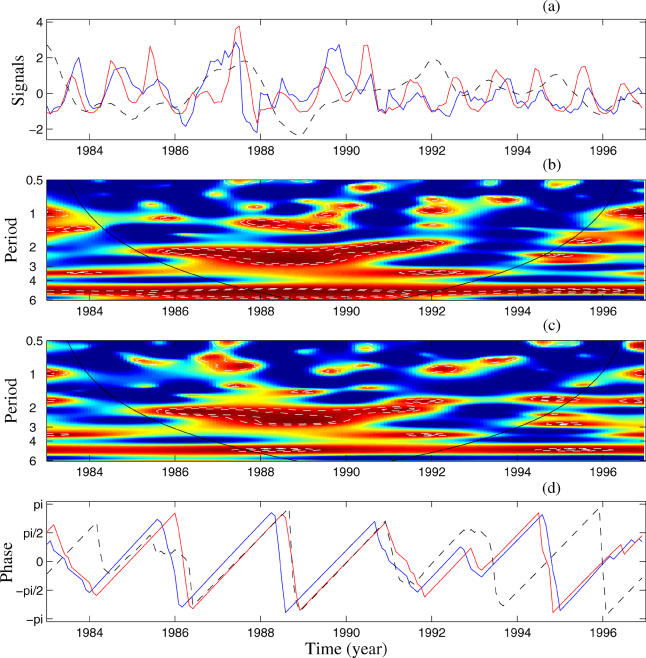
Association between Dengue in Bangkok and in the Rest of Thailand with El Niño Based on Wavelet Analysis (A) Bangkok dengue incidence (blue line), Thailand dengue incidence (red line), and Nino 3 index (black dashed line). The incidence series are square root transformed, and all series are normalised. (B) Wavelet coherence between dengue in Bangkok and Nino 3, computed using the Morlet wavelet function. The colours code for power values from dark blue for low coherence to dark red for high coherence. The nested white dashed lines show the α = 5% and α = 10% significance levels computed based on 1,000 bootstrapped series. The cone of influence indicates the region not influenced by edge effects. (C) Wavelet coherence between dengue incidence in the rest of Thailand and Nino 3. Colours as in (B). (D) Phases of time series (colours as in [A]) computed in the 2–3-y periodic band.

The oscillations of the dengue incidence time series are dominated by the annual mode of oscillation, and the El Niño is dominated by the 4–6-y components. Nevertheless, these time series have a statistically significant common mode of oscillation around a period of 2–3 y (see Figure S1). Different temporal associations between dengue and El Niño are seen in Bangkok and in the rest of Thailand (Figure 1; see also Figure S2). In each case, the wavelet analysis shows a main region of high and significant coherence for the 2–3-y periodic mode, between 1986–1992 (Figure 1B and 1C). In Bangkok, increases in dengue incidence precede changes in El Niño by several months, while for the rest of Thailand average monthly dengue incidence is perfectly in phase with El Niño (Figure 1D).

**Figure 2:**
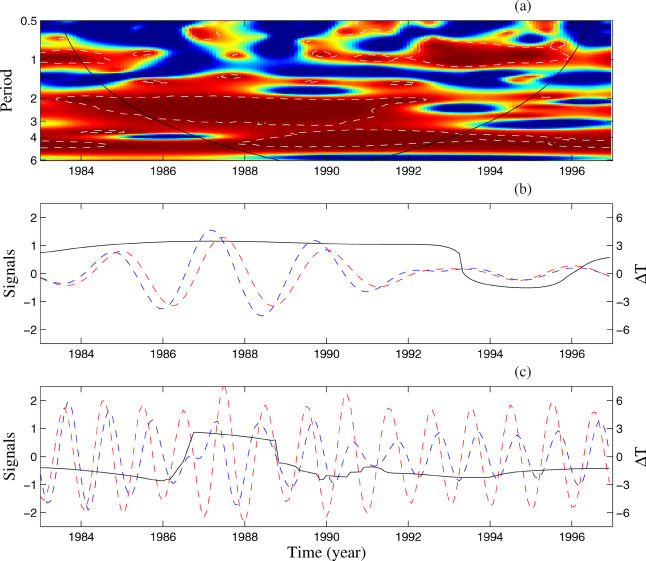
Synchronisation between Dengue Incidence in Bangkok and in the Rest of Thailand The incidence series are square root transformed, and all series are normalised. (A) Wavelet coherence computed based on the Morlet wavelet function between dengue incidence in Bangkok and in the rest of Thailand; colours as in Figure 1B. The white dashed lines show the α = 5% significance level computed based on 1,000 bootstrapped series. (B) Oscillating components computed with the wavelet transform in the 2–3-y period band (colours as in Figure 1A). (C) Oscillating components computed with the wavelet transform in the 0.8–1.2-y period band (colours as in Figure 1A). In (B) and (C) the black line shows the time evolution of the instantaneous time delay in months (Δ*T*) between the oscillating components of the two incidence time series.

The delay between dengue incidence in Bangkok and in the rest of Thailand led us to analyse the synchrony in these data using a wavelet approach (Figure 2). This analysis shows three main regions of high and significant coherence (Figure 2A). The first one is for the 2–3-y periodic band for the time period 1985–1991, the second is for the 1-y bands for 1983–1984 and for 1992–1996, and the last is for the 5-y band after 1988. This last region must be interpreted cautiously because of the short length of the time series. We also analysed the phases (not shown here) and evolution of periodic components in the 2–3-y and the 1-y bands for dengue in Bangkok and in the rest of Thailand (Figure 2B and 2C). The two incidence series are phase locked with a mean delay of 3 mo in the 2–3-y band, but only within the period of high coherence with El Niño oscillations: 1984–1992. During this time period, the major part of the variance of the dengue time series is for this 2–3-y oscillating mode (see Figure S1). For 1983–1985 and 1991–1997, dengue incidence in Bangkok follows the incidence in the remainder of Thailand with an average delay of 1 mo (Figure 2C). In these years, as the 2–3-y mode is not dominant (see Figure S1), phase locking is seen only in the 1-y (seasonal) band.

This analysis confirms that there is synchrony between DHF incidence in Bangkok and the remainder of Thailand in the 2–3-y periodic band, as recently reported [15]. However, the present findings show that this synchrony is transient and appears to be influenced by El Niño.

**Figure 3:**
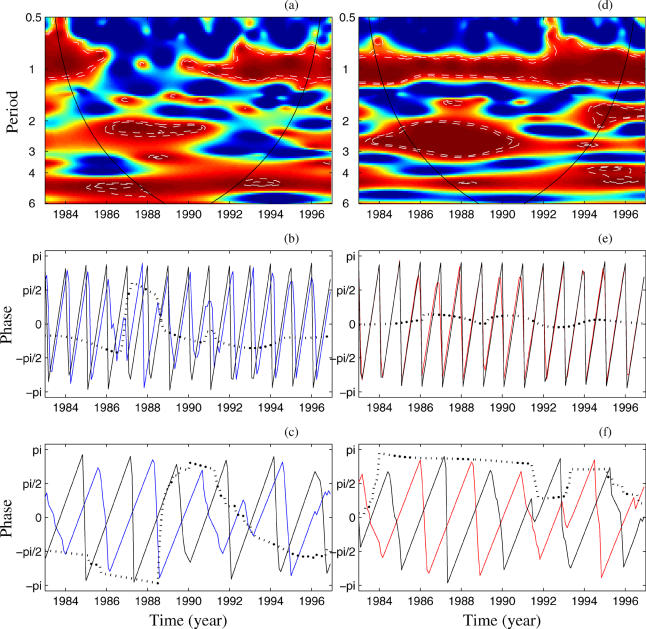
Association between Precipitation and Dengue Incidence For precipitation, gridded data [22] spatially averaged over rectangular areas representing Bangkok and the rest of Thailand using the IRI climate data library (http://ingrid.ldgo.columbia.edu/SOURCES/UEA/CRU/New/CRU05/monthly/) are used. The incidence series are square root transformed, and all series are normalised. The left part of the figure concerns Bangkok and the right part the rest of Thailand. On phase graphs, colours are as in Figure 1, and the dotted lines are for the phase difference between the considered series. (A) and (D) Wavelet coherence (see Figure 1B). (B) and (E) Phase evolutions of the considered series computed with the wavelet transform in the 0.8–1.2-y period band. (C) and (F) Phase evolutions computed in the 2–3-y period band.

In an effort to further understand this relationship, we analysed spatially averaged estimates of rainfall and temperature by month for Bangkok and for the rest of Thailand. We first focused on the link between El Niño and local climatic variables and found significant coherences in the 2–3-y periodic band only around the time period 1985–1992 in Bangkok, whereas this link is more constant throughout the study period for the remainder of Thailand (see also Figure S3). There is a highly significant coherence between the yearly components of DHF and rainfall (Figure 3A–3D). For this seasonal mode, DHF incidence and rainfall are phase locked in most of the country (Figure 3E). However, in Bangkok, the seasonal pattern of DHF incidence usually follows the seasonal peak of rainfall after a short lag time (Figure 3B). In Bangkok, in the time period 1986–1991, this association is replaced by a strong coherence in the 2–3-y band (Figure 3A). This coherence is also present for the rest of Thailand (Figure 3D), and for this mode, during the period of strong coherence, the dynamics are out of phase (Figure 3C–3F). A similar but weaker pattern of associations was observed for temperature (see also Figure S4).

## Discussion

These results provide several pieces of evidence for a complex, nonstationary relationship between El Niño, climatic variables, and DHF incidence. We have demonstrated a significant association between El Niño, climate variables, and DHF incidence for Bangkok and for the rest of Thailand. Our findings suggest that relationships between DHF and climate have a major influence on the previously reported synchrony of DHF epidemics [15].

In Bangkok, the association between DHF and climate occurs in two mutually exclusive modes, a yearly mode and a 2–3-y mode. The observed association between DHF and El Niño in the 2–3-y periodic mode coincides with the occurrence of high synchrony of DHF throughout Thailand initiated in the capital city, Bangkok. If the association in the yearly periodic mode becomes dominant, the synchrony of DHF dynamics initiated in Bangkok collapses and both the dynamics and the synchrony are dominated by the seasonal components (see Figure 2). In the rest of Thailand, the 2–3-y mode is never completely dominant and the seasonal mode persists throughout the dataset.

The complexity of the link between dengue dynamics and climate is emphasised by the positive correlations in the seasonal mode and negative correlations in the 2–3-y periodic mode. The results are consistent with the observation that, in most countries, dengue is most prevalent in the wet season, yet on an interannual scale, dengue epidemics have also been associated with drought [13]. In countries with high rainfall, drought can cause normally fast-flowing rivers to recede into a series of stagnant pools, ideal for mosquito breeding. On the other hand, in the Pacific Islands, dengue epidemics tend to occur during La Niña events, which are associated with conditions warmer and wetter than normal in most islands [11]. Dengue and climate might be driven by temperature, rather than rainfall.

Dengue in Bangkok seems to precede the oscillations of the Nino 3 index. This may reflect the timing of relationships between El Niño and climate. Another potential explanation could be a nonlinear or a threshold effect between large-scale phenomena and local dengue dynamics, as previously suggested for cholera [23]. The oscillations of the epidemics would be produced by local climatic phenomena generated before the maxima of the large-scale phenomena.

Alternatively, dengue epidemics might start in a nearby country where the effect of El Niño is more pronounced. Movement of infected vectors or travellers between countries could lead to propagation of the disease in synchrony with El Niño [12].

Whether the underlying climatic influence is local or regional, our findings suggest a biologically plausible mechanism for the recently reported synchronous dynamics of DHF in Thailand in the years 1985–1991. We hypothesise that under certain conditions, interannual variation in local or regional climate linked to El Niño may act as a pacemaker, modulating both the temporal dynamics and the spatial synchrony of DHF in a travelling wave.

These findings do not exclude an important role for other factors, such as intrinsic disease dynamics, in explaining patterns of dengue incidence in Thailand [24]. A previous study reported no apparent relationship between dengue and interannual climate in Bangkok between 1966 and 1998 [14]. However, in this work the authors [14] used spectral density analysis, which is not sensitive to nonstationary effects. Conventional statistical methods may fail to reveal a strong relationship between climate and a health outcome when discontinuous associations are present. The association between dengue and climate reported here is strong but transient. Nonstationarity can make it difficult to demonstrate even strong climate–health relationships. This has been reported by Rodó et al. [23] in the case of cholera epidemics. They have shown that the association between El Niño and cholera prevalence in Bangladesh is strong but transient. In the earlier part of the century, periodic components of cholera and El Niño were not associated, whereas late in the century (1980–2001) the relationship between these components was strong.

There is considerable interest in the role played by climate variability as a factor driving diseases [2,3,4,5,6,7,23,24,25]. Wavelet analyses can reveal transient population synchrony as well as long-term climate–health relationships. Future studies should use this approach to examine relationships between climate and dengue fever on regional and global scales, and attempt to identify the geographical location of the hypothesised pacemaker.

## Supporting Information

Figure S1 Wavelet Transform of the Dengue Incidence and Nino 3 Time Series The incidence series are square root transformed, and all series are normalised. The dashed lines, white or black, show the α = 5% significant levels computed based on 1,000 bootstrapped series. On the scalograms in (A), (C), and (E), the cone of influence, which indicates the region not influenced by edge effects, is also shown.
(A) Wavelet power spectrum (*S_x_*(*f*, τ)) of dengue incidence in Bangkok. The colours code for power values from dark blue for low values to dark red for high values.
(B) The average wavelet spectrum of the time series.
(C and D) As in (A and B) but for the time series of dengue incidence in the rest of Thailand.
(E and F) As in (A and B) but for the time series of the Nino 3 index.
(358 KB EPS).

Figure S2 Association between Dengue in Bangkok and in the Rest of Thailand with El Niño Based on Wavelet Analysis of the Southern Oscillation Index (72 KB EPS).

Figure S3 Associations between Climate and El Niño For El Niño, the Nino 3 index is employed, and for climatic variables, gridded data [22] spatially averaged over rectangular areas representing Bangkok and the rest of Thailand using the IRI climate data library (http://ingrid.ldgo.columbia.edu/SOURCES/.UEA/.CRU/.New/.CRU05/.monthly/) are used. The left part of the figure concerns Bangkok and the right part the rest of Thailand. The seasonal components of the time series have been removed by filtering with a low-pass filter and a cutoff at 15 mo, and all series are normalised. (A–D) are related to rainfall and (E–H) to temperature.
(A), (C), (E), and (G) Wavelet coherence (see Figure 1.).
(B), (D), (F), and (H) Phase evolutions of the considered series computed with the wavelet transform in the 2–3-y period band. On phase graphs, colours are as in Figure 1, and the dotted lines are for the phase difference between the considered series.
(499 KB EPS).

Figure S4 Association between Temperature and Dengue Incidence For temperature, gridded data [22] spatially averaged over rectangular areas representing Bangkok and the rest of Thailand using the IRI climate data library (http://ingrid.ldgo.columbia.edu/SOURCES/.UEA/.CRU/.New/.CRU05/.monthly/) are used. The incidence series are square root transformed, and all series are normalised. The left part of the figure concerns Bangkok and the right part the rest of Thailand. On phase graphs, colours are as in Figure 1, and the dotted lines are for the phase difference between the considered series.
(A) and (D) Wavelet coherence (see Figure 1B).
(B) and (E) Phase evolutions of the considered series computed with the wavelet transform in the 0.8–1.2-y period band.
(C) and (F) Phase evolutions computed in the 2–3-y period band.
(270 KB EPS).

**Patient Summary**

**Background**

Many things interact to determine when epidemics of disease occur. Some of these factors are due to the disease-causing agent itself or what carries it; other factors include climate, both local and over a larger region. Dengue fever, caused by a virus and transmitted by a mosquito, has a very complex pattern of epidemics.

**What Did the Researchers Do?**

They examined the pattern of dengue outbreaks, specifically the most serious form of dengue, dengue hemorrhagic fever, in the 72 provinces of Thailand between 1983 and 1997 and also looked at climate patterns, especially those caused by El Niño.

They found that though El Niño was associated with some specific disease outbreaks between 1986 and 1992, it was not associated with all of them, and for the remaining outbreaks other, more local factors were likely to be more important.

**What Do These Findings Mean?**

They provide more information about how dengue epidemics start and spread. They may be useful for those who plan public health measures in affected countries.

**Where Can I Get More Information?**

The United States Centers for Disease Control and Prevention has a Web page on dengue: http://www.cdc.gov/ncidod/dvbid/dengue/

The World Health Organization also provides information: http://www.who.int/mediacentre/factsheets/fs117/en/

MedlinePlus has a Web page aimed specifically at patients with dengue: http://www.nlm.nih.gov/medlineplus/ency/article/001374.htm

## Abbreviations

DHF: dengue haemorrhagic fever
